# Monitoring cell productivity for the production of recombinant proteins by flow cytometry: An effective application using the cold capture assay

**DOI:** 10.1002/elsc.202000049

**Published:** 2021-01-06

**Authors:** Katharina V. Meyer, Ina G. Siller, Jana Schellenberg, Alina Gonzalez Salcedo, Dörte Solle, Jens Matuszczyk, Thomas Scheper, Janina Bahnemann

**Affiliations:** ^1^ Institute of Technical Chemistry Leibniz University Hannover Hannover Germany; ^2^ Sartorius Stedim Biotech GmbH Göttingen Germany

**Keywords:** CHO cells, mammalian cell culture, monoclonal antibody production, secretion assay, specific cell productivity

## Abstract

Due to the increasing economic and social relevance of biotherapeutics, their production processes are continually being reconsidered and reoptimized in an effort to secure higher product concentrations and qualities. Monitoring the productivity of cultured cells is therefore a critically important part of the cultivation process. Traditionally, this is achieved by determining the overall product titer by high performance liquid chromatography (HPLC), and then calculating the specific cell productivity based on this titer and an associated viable cell density. Unfortunately, this process is typically time‐consuming and laborious. In this study, the productivity of Chinese Hamster Ovary (CHO) cells expressing a monoclonal antibody was analyzed over the course of the cultivation process. In addition to calculating the specific cell productivity based on the traditional product titer determined by HPLC analysis, culture productivity of single cells was also analyzed via flow cytometry using a cold capture assay. The cold capture assay is a cell surface labelling technique described by Brezinsky et al., which allows for the visualization of a product on the surface of the producing cell. The cell productivity results obtained via HPLC and the results of cold capture assay remained in great accordance over the whole cultivation process. Accordingly, our study demonstrates that the cold capture assay offers an interesting, comparatively time‐effective, and potentially cheaper alternative for monitoring the productivity of a cell culture.

**Abbreviations**
a. u.: arbitrary units
APC: allophycocyanin
CHO: Chinese Hamster Ovary
CVCT: cumulative viable cell time
ELISA: enzyme‐linked immunosorbent assay
HPLC: high performance liquid chromatography
mAb: monoclonal antibody
MFI: mean fluorescence intensity
PBS: phosphate buffered saline
qP: specific cell productivity
SF: shake flask
VCD: viable cell density

1

Biotherapeutics derived from mammalian cell cultures are constantly gaining importance within the pharmaceutical industry [2, 3]. The increased demand for these products is continually driving a push for ever more efficient production processes [4, 5, 6]. Product yield of recombinant protein is essentially determined by two factors: The count of viable cells and the specific productivity of the cells during the cultivation process [7]. As a result, carefully and accurately monitoring these two parameters is of importance in developing a high‐yielding process. The most common method for achieving this is to determine product titer via either a high performance liquid chromatography (HPLC) analysis [7, 8, 9] or an enzyme‐linked immunosorbent assay (ELISA) [2, 10, 11, 12]. However, both of these methods are regrettably time‐consuming and laborious. For example, ELISA protocols involve several incubation steps and the addition of different reagents, which have to be handled manually [2, 12]. HPLC systems require high maintenance and the measurements themselves are rather lengthy [13].

In this study, the productivity of Chinese Hamster Ovary (CHO) cells expressing a monoclonal antibody (mAb) was analyzed over the course of the cultivation process. In addition to calculating the specific cell productivity based on the product titer determined by HPLC analysis, the productivity was also analyzed by flow cytometry using the cold capture assay. A schematic overview of the analysis is shown in Figure 1. The cold capture assay was first described by Brezinsky et al. [1] and constitutes a cell surface labelling technique using fluorescently labelled antibodies that bind to the secreted target proteins. This method is based on the fact that proteins for secretion are transported from the Golgi apparatus to the cell membrane via vesicles that join the plasma membrane and so release their content into the extracellular environment [1, 4]. Therefore proteins secreted by a cell can be visualized on its surface [1, 4]. This method has previously been used to enrich high producing cells by fluorescent activated cell sorting [1, 2, 14, 15], but to the best of the authors’ knowledge, this study represents the first time that it has been reported as a method of successfully monitoring the productivity of cultured cells over the time course of the cultivation process.

**FIGURE 1:**
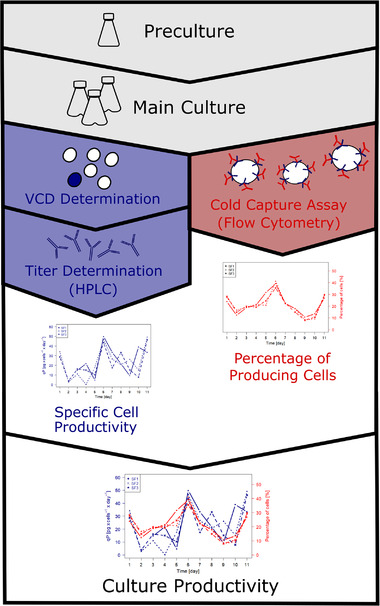
Flow chart of the performed experiments. The productivity of the cultured cells was analyzed based on VCD and product titer as well as by flow cytometry

A Cellca CHO DG44 cell line (Sartorius, DE) expressing a human IgG1 mAb was used for the experiments. The cultivation process started by conducting a seed culture. In brief, the cryoconserved cells were thawed and passaged five times, every 3‐4 days. For all passages single‐use 0.5 L Erlenmeyer Shake Flasks (Corning, USA) with 150 mL pre‐warmed cell line associated stock culture medium (Sartorius, DE) were used. The cells were incubated (HeracellTM 240, Thermo Fisher Scientific, USA) at a temperature of 36.8°C, 85% humidity, a pCO_2_ of 7.5% and a shaking rate of 120 rpm with an orbital diameter of 19 mm. The resulting main culture was then inoculated with 0.3 × 10^6^ cells·mL^−1^ in three single‐use 0.125 L Erlenmeyer Shake Flasks (Corning, USA), with 25 mL pre‐warmed cell line associated basal medium for production (Sartorius, DE). The main culture was run as a fed‐batch process, starting with a batch‐phase of 72 h, for 12 cultivation days in total. Starting from day 5 of this process, a glucose solution (Sigma Aldrich, USA) was supplied in addition to the cell line associated daily feed (Sartorius, DE) once the glucose level dropped below 5 g·L^‐1^. The main culture was kept at the same culture conditions described above for the seed culture, and all used feeds and media were chemically defined.

**PRACTICAL APPLICATION**

Monitoring the productivity of cultured cells is an important tool for cultivation process optimization. This is frequently be done by determining the product titer using HPLC analysis which can be a time‐consuming and laborious process. The cold capture assay offers an interesting alternative, through which researchers can potentially monitor cell culture productivity using flow cytometry. This method offers segregated data about all populations instead of integral data such as the overall volumetric titer concentrations. Based on this high producer clones can be separated or the process conditions can be adjusted to a high production of all cells resulting in high titer concentrations.

Throughout this experiments, a sample was taken daily from each shake flask (SF 1‐3). Viable cell density (VCD) and viability of the culture were analyzed using a trypan blue assay–based cell counter (Cedex HiRes, Roche, CHE). The titer of the mAb in the cell culture supernatant was determined by a HPLC (Chromaster VWR International GmbH, USA) method with a size exclusion column (Yarra™ 3 μm SEC 3000 OOH‐4513‐KO 300 x 7.8 mm, Phenomenex Inc., USA) controlled by the HPLC‐Software Open Lab Control Panel (Agilent Technologies, USA). The method was 20 min long with a flow rate of 1 mL·min^‐1^, an oven temperature of 25°C and a sample injection volume of 5 μL. The product peak was measured using a diode array detector (280 nm), and the product concentration was determined using a standard curve prepared with known product concentrations. For the HPLC analysis a buffer (pH 6.6) containing 100 mL of 1 M sodium sulfate (Carl Roth GmbH & Co. KG, DE), 50 mL 1 M sodium dihydrogen phosphate (Sigma‐Aldrich, Merck KgaA, DE), 50 mL 1 M disodium hydrogen phosphate (Sigma‐Aldrich, Merck KgaA, DE) and 800 mL ddH2O (Arium^®^ Sartorius, DE) was used.

Figure 2 shows the VCD, viability and mAb concentration of the main culture over the course of the cultivation process. The parameters demonstrated similar development across all three shake flasks. In all three flaskes, cell viability remained above 90% up until and including day 10, and thereafter decreased to approximately 81% on the last cultivation day. As expected, the maximal VCD was reached on day 8 (SF1: 12.44 × 10^6^ cells·mL^‐1^, SF2: 14.99 × 10^6^ cells·mL^‐1^, SF3: 14.75 × 10^6^ cells·mL^‐1^). Until day 5, only a slight increase in product titer could be observed, while thereafter the titer showed a larger increase. The highest titer was reached on day 11 in shake flasks 1 and 3 (SF1: 2.1 g·L^‐1^, SF3: 2.5 g·L^‐1^), and on day 12 in shake flask 2 (SF2: 2.4 g·L^‐1^). Shake flask 1 and Shake flask 3 also showed a decrease in product titer on day 12, at the end of the cultivation process. This decrease could have been caused by the decreasing viability at the end of the cultivation. However, it bears noting that the viability decreased similarly across all three shake flasks while the decrease of the mAb titer is only observed for two of the three shake flasks. VCD and product titer were used to calculate the specific productivity of the cells. Since cell viability and mAb titer decreased notably on the last day of cultivation, specific cell productivity was only calculated up until day 11. The specific cell productivity (qP) [pg·cell^‐1^·day^‐1^] was calculated employing the following Equation 1 as described in Edros, McDonnell and Al‐Rubea [10]. Here, mAb represents the antibody concentration at a particular time while the cumulative viable cell time (CVCT) is calculated employing Equation 2, where x_n_ is the VCD [10^6^ cells·mL^‐1^] at a particular time t_n_ [day] and x_n+1_ the VCD [10^6^ cells·mL^‐1^] after the elapsed time t_n+1_ [day].

(1) $$qP=\frac{mAb_{n+1}-mAb_{n}}{CVCT}$$

(2) $$CVCT=\frac{x_{n+1}+x_{n}}{2}\times \left(t_{n+1}-t_{n}\right)$$

**FIGURE 2:**
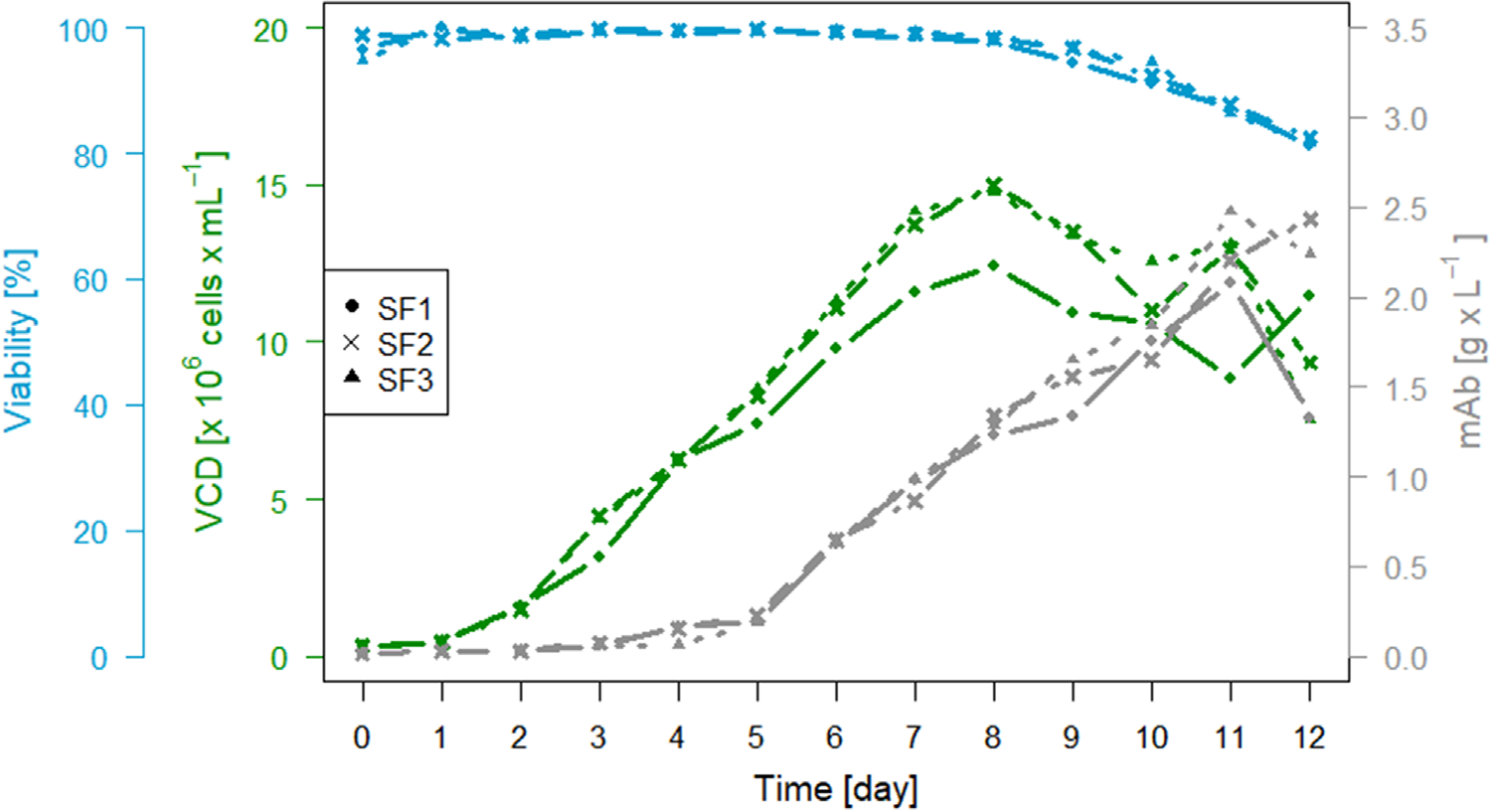
VCD, viability and mAb concentration during the cultivation in all shake flasks (SF1‐3)

The calculated specific cell productivity of the main culture is shown in Figure 3 in blue. Its time course was similar for all three shake flasks. A maximum of the specific cell productivity was also observed across all three shake flasks on day 6 of the cultivation (SF1: 49.71 pg·cell^‐1^·day^‐1^, SF2: 43.33 pg·cell^‐1^·day^‐1^, SF3: 46.42 pg·cell^‐1^·day^‐1^). This maximum is in accordance with the already mentioned great increase of the product titer from day 5 to day 6. The other process related maximum of the specific cell productivity towards the end of the cultivation process (day 10/11) could also be observed.

**FIGURE 3:**
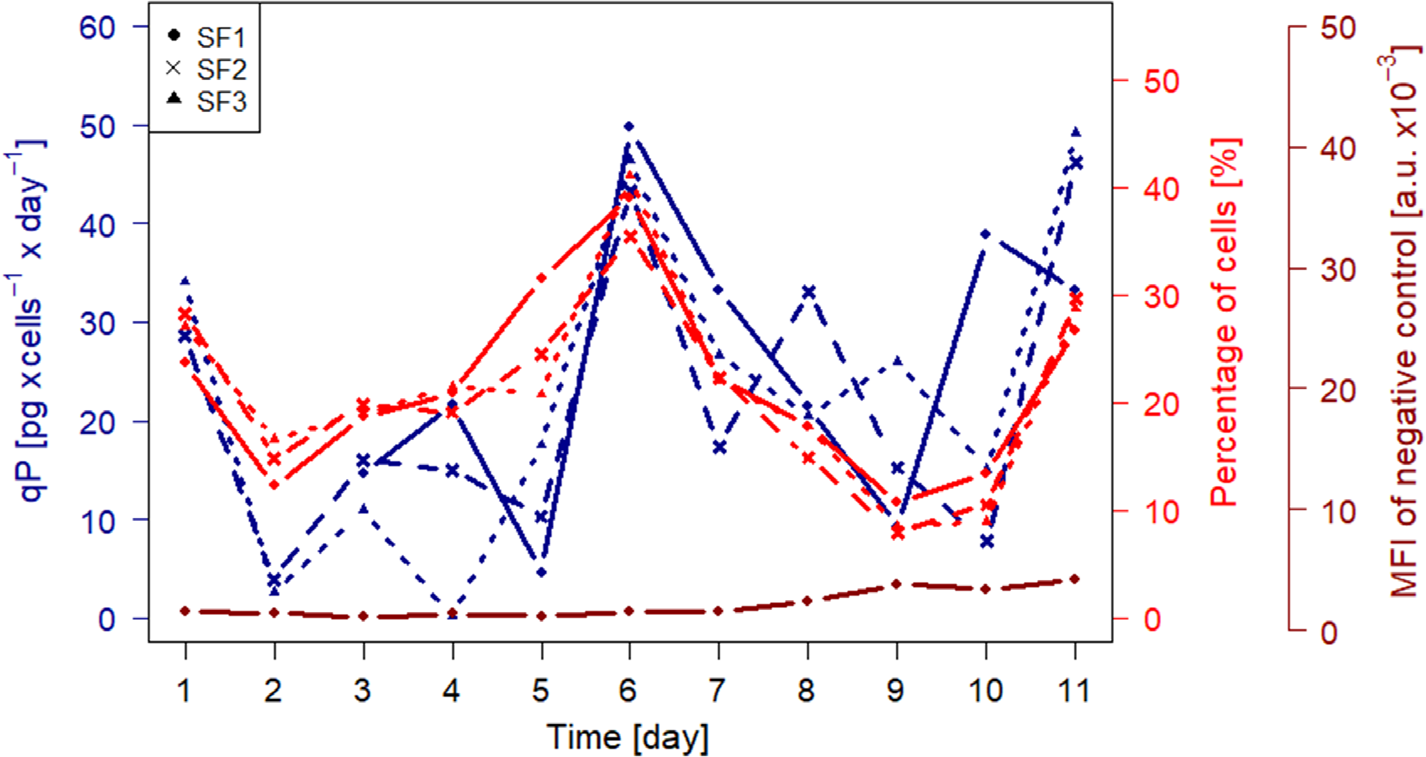
Specific cell productivity of the main culture calculated according to Equation 1 (blue) and percentage of cells in the cold capture assay (bright red) for all three shake flasks (SF1‐3). As a scale of variation for the cold capture assay, the mean fluorescence intensity (MFI) in arbitrary units (a. u.) of the negative control is shown as well (dark red)

In addition to calculating the specific cell productivity based on the product titer determined by HPLC analysis and VCD, the productivity of the main culture was also analyzed by flow cytometry using the cold capture assay. In this study a staining method based on the information provided by Brezinsky et al. [1] and Pichler et al. [4] regarding the cold capture assay was used and the manufacturer specifications of the utilized anti‐human‐IgG1‐allophycocyanin (APC) antibody (Miltenyi Biotec, DE) were considered. Briefly, the cell suspension was centrifuged at room temperature for 5 min at 300 × g (Centrifuge 5702, Eppendorf, DE) and a pellet of up to 10^6^ cells was then resuspended in 98 μL 4°C cold phosphate buffered saline (PBS). Subsequently, 2 μL of the staining antibody were added and the suspension was then mixed by careful pipetting and kept in the dark at 4°C for 15 min. Afterwards, these cells were washed using 1 mL of 4°C cold PBS and centrifuged at 4°C for 5 min at 300 × g (Heraeus Megafuge 8R Thermo Fisher Scientific, USA). The supernatant was thereafter discarded, and the cell pellet was resuspended in 500 μL 4°C cold PBS and kept on ice. A BD Accuri™ C6 (Becton Dickinson, USA) was used to conduct the flow cytometry analysis. For all samples, at least 10,000 events were measured and data analysis was run using BD Accuri™ C6 software (Becton Dickinson, USA), R [16] and TinnR [17]. Unstained Cellca CHO DG44 cells were used as a negative control. In further experiments stained non‐producing Cellca CHO DG44 cells should be included as an additional control. Intact cells were gated using the forward scatter‐area vs. side scatter‐area signal and a doublet discrimination was conducted. The corresponding red fluorescence signal of the APC linked to the anti‐human‐IgG1 antibody has an excitation maximum of 650 nm and an emission maximum of 660 nm [18] and was captured using appropriate filter settings. Finally, the percentage of events showing a higher red fluorescent signal in the side scatter‐area vs. APC‐area plot than the negative control was analyzed.

The results of the cold capture assay are shown in Figure 3 in bright red. The time course of the signal obtained in the cold capture assay was similar for all three shake flasks. A maximum could be observed on day 6 of the cultivation (SF1: 39.04%, SF2: 35.48%, SF3: 41.04%), the same day that the calculated specific cell productivity reached its apex. Similar to the specific cell productivity, the signal obtained in the cold capture assay also showed an increase towards the end of the cultivation. Further experiments with a larger sample size could be considered for a statistical analysis of the correlation between the specific cell productivity and the results of the cold capture assay. Overall, the specific cell productivity determined via HPLC and the results of the cold capture assay remained in great accordance over the whole cultivation process. When comparing the two methods, however, it should be noted that the cell productivity determined by HPLC analysis is averaged over the entire cell population (integral data), whereas in the cold capture assay using flow cytometry the cells are analyzed individually (segregated data). Thus, the latter method offers a great potential for process optimization, since by observing individual cells, high producers can be identified and selected. It should also be considered that the calculation of the specific productivity via HPLC analysis is dependent on the product titer as well as the VCD. As a result, this method requires the input of data obtained from two instruments, while the cold capture assay results are based on data from only one instrument. Furthermore, the specific cell productivity calculated with the help of HPLC results is influenced by values previously obtained, since analysis of the supernatant inherently reflects previous processes and already secreted proteins. In contrast, the signal obtained by cold capture assay shows the current state of the cells. This may prove beneficial for monitoring quick changes of productivity during a cultivation process. The results demonstrate that the use of flow cytometry to monitor cell productivity therefore delivers a comparatively fast and reliable analysis, and consequently allows for comparatively quicker adaptations of ongoing production processes. Especially in situations where the titer determination via HPLC is complicated for a specific product, the cold capture assay offers an easier to use and more time efficient method to secure an overview of the productivity of a conducted culture process. Moreover, with only limited additional effort, the analysis of other culture‐relevant parameters is also possible using this method for example, observing of the apoptosis/necrosis status of a culture. In summary, the cold capture assay offers an excellent alternative to more traditional methods for monitoring productivity during a cultivation process and shows great potential for further process optimization.

## CONFLICT OF INTEREST

The authors have declared no conflict of interest.

## Data Availability

The data that support the findings of this study are available from the corresponding author upon reasonable request.
